# Putrescine eases saline stress by regulating biochemicals, antioxidative enzymes, and osmolyte balance in hydroponic strawberries (cv. Albion)

**DOI:** 10.1111/ppl.70259

**Published:** 2025-05-09

**Authors:** Ferhad Muradoğlu, Şeyma Batur, Mirmahmud Hasanov, Emrah Güler

**Affiliations:** ^1^ Department of Horticulture Faculty of Agriculture, Bolu Abant Izzet Baysal University Bolu Türkiye; ^2^ Institute of Graduate Education, Faculty of Agriculture Bolu Abant Izzet Baysal University Bolu Türkiye

## Abstract

Salinity is a significant abiotic stress factor that causes considerable damage to many plants through various mechanisms. In this study, the ameliorative effect of putrescine (100, 150, and 200 ppm) on salinity stress (1 g L^−1^ NaCl) was investigated in strawberry cv. Albion grown in hydroponic culture. Results showed that putrescine provided root development comparable to control under salt stress. Chlorophyll a and b levels were slightly enhanced by putrescine, while the Chlorophyll a/b ratio was significantly improved. The total phenolics, carbohydrates, anthocyanin, and protein were also raised by putrescine treatments under saline conditions. The antioxidant mechanism was also fortified by putrescine treatment, evidenced by the increased DPPH scavenging and the rise in the antioxidant enzymes, SOD and CAT. The oxidative substances, particularly MDA, notably decreased by rising putrescine doses. Moreover, putrescine treatments positively regulated root‐to‐shoot osmolyte homeostasis with a diverse effect mechanism according to applied doses. Overall, this study provides a comprehensive insight into the effects of putrescine on the saline stress mechanism in strawberries and suggests it as a favourable biogenic agent.

## 1 INTRODUCTION

Strawberry, in the group of berry fruits, is a type of fruit that is not a true fruit and is formed from the flower base. The strawberry fruits have many areas of use and are generally used in table, jam, dried fruit, marmalade, and fruit juice production (Abouelenein et al., 2021). Today, *Fragaria* × *ananassa* strawberries are the most commercially grown strawberry species. The day‐neutral or short‐day varieties are preferred more among the commercially grown strawberry varieties, and long‐day varieties are reported to produce fruit continuously (Hernández‐Martínez et al., 2023). Strawberries have been cultivated since ancient times and are widely preferred in areas suitable for agriculture due to their high adaptability. For this reason, strawberry cultivation has rapidly increased in recent years. The rapid profit return of the investments in strawberry cultivation highly increased the demand for strawberry growing. (Erdem Öztürk & Çekiç, 2017; Zacharaki et al., 2024).

One of the primary physiological responses of strawberries to stress is the accumulation of osmoprotectants, such as proline. Under drought stress, proline levels increase as a mechanism for osmotic adjustment, helping to maintain cell turgor and protect cellular structures from damage (Sun et al., 2015; Thokchom & Hazarika, 2024). This accumulation is often accompanied by increased activity of antioxidant enzymes, which mitigate oxidative stress caused by reactive oxygen species (ROS) generated during adverse conditions (Doğan et al., 2022; Wang et al., 2022). Studies have shown that drought stress leads to elevated levels of malondialdehyde (MDA) and hydrogen peroxide (H_2_O_2_), indicating oxidative damage in strawberry leaves (Muradoglu et al., 2015; Karakaş et al., 2021). Muradoğlu et al. (2016) noted a significant enhancement of MDA and antioxidant enzymes (SOD, CAT, APX) in both leaves and roots of strawberries under lead stress. The application of exogenous compounds like 5‐aminolevulinic acid has been reported to enhance the tolerance of strawberries to osmotic stress, potentially by modulating proline metabolism and antioxidant responses (Cai et al., 2019). Moreover, Muradoğlu et al. (2020) reported positive effects of exogenous application of methyl jasmonate on growth parameters under heavy metal (cadmium) stress.

Salinity stress is a significant challenge for strawberry cultivation, as it adversely affects various physiological and biochemical processes, ultimately leading to reduced growth and fruit quality. Strawberry plants are particularly sensitive to salinity, which can cause leaf necrosis, nutrient imbalances, and reduced photosynthetic efficiency (Karlıdağ et al., 2013; Moneim et al., 2021; Jamali et al., 2016). Salt stress induces oxidative damage, characterized by increased MDA levels and reduced chlorophyll content, which adversely impacts photosynthesis (Hnilickova et al., 2021; Karakaş et al., 2021). The physiological responses to salt stress include alterations in stomatal conductance and leaf relative water content, leading to reduced gas exchange and photosynthetic efficiency (Doğan et al., 2022). The physiological responses of strawberries to salinity stress include decreased chlorophyll content, reduced dry matter accumulation, and impaired water uptake, which collectively contribute to diminished plant vigour and yield (Aras & Eşitken, 2019; Ghaderi et al., 2018).

Putrescine, a biogenic amine classified as a polyamine, has emerged as a significant player in plant stress responses, particularly under abiotic stress conditions such as drought, salinity, and temperature extremes (Hossain et al., 2022). Its function as a stress reducer can be explained by several mechanisms, including its ability to stabilize cellular structures, scavenge reactive oxygen species, and modulate metabolic pathways (Rajkumari et al., 2023). One of the primary functions of putrescine in stress tolerance is its involvement in ROS scavenging. Under stress conditions, plants often experience oxidative stress due to the accumulation of ROS, which can damage cellular components (Anam et al., 2024). Studies have shown that putrescine can act as a direct radical scavenger, thereby mitigating oxidative damage. Inal et al. (2022) demonstrated that putrescine reduces oxidative stress under cold conditions by enhancing the activity of antioxidant enzymes, which allows for better management of oxidative stress. Similarly, Mohammadi‐Cheraghabadi et al. (2021) highlighted that putrescine enhances enzymatic antioxidant activity, which is crucial for maintaining plasma membrane integrity during water deficit stress. This protective role is further supported by findings from Gong et al. (2015), who noted that putrescine accumulation is associated with physiological changes that enhance drought tolerance in plants.

This study investigated the efficiency of putrescine in reducing salt stress in strawberry plants grown in hydroponic culture.

## 2 MATERIALS AND METHODS

### 2.1 Plant Material

In this study, Albion strawberry variety was used as plant material. Albion is a strawberry variety developed by the University of California in 1999 through the hybridization of Diamante and Cal 94.16–1. It is classified as a day‐neutral variety, meaning its fruiting is not dependent on light conditions. The fruit of the Albion variety is long, conical, and quite symmetrical in shape. Both the inside and outside of the fruit are dark, and it is known for its sweet taste. The phenotypic characteristics of Albion can vary depending on the growing conditions. Typically, the length of Albion strawberries ranges from approximately 210 to 270 mm, with a fruit diameter of about 55 to 75 mm. According to Shaw and Larson (2006), the yield for this variety is around 2417 grams (g) per plant, with each fruit weighing approximately 33 g.

### 2.2 Methods

This study was conducted in the aquaculture facilities of the Horticulture Laboratory and the climate chamber at the Faculty of Agriculture, Bolu Abant Izzet Baysal University. Frigo seedlings of the Albion variety were used as study material. The research focused on the effects of different external doses of putrescine (100, 150, and 200 ppm) on salt stress in Albion seedlings subjected to 1000 mg L^−1^ of salt stress. Physiological and enzyme measurements were performed in the laboratories. The necessary extracts for nutrient element analysis were prepared in the Physical Analysis Laboratory of the Seed Science and Technology Department at the same faculty.

#### 2.2.1 Plant growing and treatment

The experiment was conducted in a climate chamber and through hydroponic culture. The hydroponic culture was implemented in plastic tubs measuring 46 × 42 × 15 cm. Hard styrofoam was used to hold the seedlings, with appropriately sized holes created in the styrofoam for each seedling. After cleaning the roots, the seedlings were placed in these holes. The roots were then immersed in the Hogland nutrient solution and positioned on the hard styrofoam tubs.

The ambient temperature in the climate chamber where the experiment was conducted was set to 22°C during the day and 18°C at night. The light duration was 16 h, and the humidity was maintained at 65–70%. A white fluorescent lamp providing a light intensity of 280 μmol m^2^ s^−1^ was used as the light source. Salt (1000 mg L^−1^) was not applied at the beginning of the experiment; instead, it was introduced with the irrigation water after the strawberry seedlings developed approximately 4–5 leaves, which occurred around 30 days into the experiment, using a Hoagland ½ nutrient solution. The salt doses were determined based on the research conducted by Yildirim et al. (2009), which examined the effects of a 40 mM NaCl concentration (approximately 2.3 g L^−1^) under soil conditions. Considering that soil has a buffering capacity, we decided to apply half of this dose to prevent excessive damage from direct contact with the water. The solution was changed every 4 to 5 days, and the same dose of NaCl concentration was added to ensure the consistency of the determined salt concentration during each change. Doses of putrescine (100, 150, and 200 ppm) were applied to the plants as foliar sprays 10 days after salt application, based on previously utilized doses in strawberries by Kuru Berk et al. (2023). The salt and putrescine treatments were conducted over four weeks, and samples were collected as soon as the experiment finished.

#### 2.2.2 Plant growth parameters

In this study, 10 plants were randomly selected for each replication from each group. The roots and leaves of these plants were separated and weighed individually using a digital scale with a sensitivity of 1 milligram to determine their fresh weights. Root lengths were measured using a ruler, while the number of roots and leaves was counted individually. The dry weights of the roots and leaves were obtained by drying the samples in an oven at 80°C for 48 h, after which they were weighed again on the digital scale.

#### 2.2.3 Chlorophyll and carotenoid analysis

The determination of chlorophyll amounts was conducted following the method outlined by Withan et al. (1971) using a spectrophotometric approach. In brief, 0.5 g of a fresh leaf sample was homogenized with 80% acetone and filtered through Whatman filter paper into 50 mL measuring flasks. The filtrates were measured at 645 and 663 nm wavelengths using a UV spectrometer (D‐LAB SPUV1100). The resulting values were then used to calculate the amounts of chlorophyll a, b, and total chlorophyll according to the formula proposed by Withan et al. (1971).

Chl A (mg g^−1^) = 12.7× (D663–2.69) × (D645) × V/1000 × W.

Chl B (mg g^−1^) = 22.91 × (D645–4.68) × (D663) × V/1000 × W.

The total carotenoid amount of the samples was determined by calculating the values obtained from the same filters at 450 nm wavelength in the UV spectrometer according to the formula below (Lichtentilaler, 1986).

Total Carotenoids (mg g^−1^) = 4.07 × A450 − (TChl).

A = Absorbance, TChl = Total chlorophyll amount.

#### 2.2.4 Antioxidant enzyme analyses

The samples collected for the study were stored in a − 80°C freezer until analysis. For the antioxidant analysis, approximately 1 g of leaf sample was crushed in a porcelain mortar using liquid nitrogen. The crushed samples were then transferred to a tube, and a solution containing 0.1 mM Na‐EDTA and 50 mM phosphate buffer (pH 7.6) was added. The mixture was homogenized using a vortex for 15 s. After homogenization, the samples were centrifuged at 12000 *g* for 15 min. The resulting supernatant was kept on ice at +4°C until the enzyme analyses were conducted. The obtained supernatant was then analyzed with a UV spectrophotometer.

The activity of ascorbate peroxidase (APX) was determined at 290 nm (E = 2.8 mM cm^−1^) following the methods outlined by Çakmak and Marschner (1992) and Çakmak (1994). To prepare the reaction medium, we combined the following components: 50 mM phosphate buffer (pH 7.6) containing 0.1 mM EDTA, 0.1 mL of 12 mM H₂O₂ with 10 mM EDTA, 0.1 mL of 0.25 mM L (−) ascorbic acid, and the enzyme extract. The total volume was adjusted to 2 mL, and ascorbate oxidation was measured spectrophotometrically at 290 nm.

Superoxide dismutase (SOD) activity was measured by evaluating the reduction of nitroblue tetrazolium chloride (NBT) by superoxide (O_2_^−^) under light, following the methods outlined by Çakmak and Marschner (1992) and Çakmak (1994). The procedure began with the addition of 50 mM phosphate buffer (pH 7.6) containing 0.1 mM Na‐EDTA. Next, 0.5 mL of 50 mM NaCO_3_ (pH 10.2), 0.5 mL of 12 mM L‐methionine, 0.5 mL of 75 μM NBT, and an enzyme extract (25–50 μL) were added. Finally, 0.5 mL of 10 μM riboflavin was included to achieve a final volume of 5 mL. To facilitate the reduction of NBT by superoxide, the samples were kept at 24°C and exposed to a light intensity of 400 μmol m^−2^ s^−1^ for 15 min. SOD activity was quantified as the amount of enzyme required to inhibit 50% of the NBT reduction rate, which was measured at a wavelength of 560 nm.

The activity of the catalase (CAT) enzyme was measured by determining the rate at which hydrogen peroxide (H_2_O_2_) is degraded, using a wavelength of 240 nm (with an extinction coefficient of 39.4 mM cm^−1^). The reaction medium was prepared by adding 0.1 mL of a 100 mM H_2_O_2_ solution, 50 mM phosphate buffer (pH 7.6), 0.1 mM EDTA, and the enzyme extract to achieve a final volume of 2 mL (Yılmaz et al., 2023).

The analysis of hydrogen peroxide (H_2_O_2_) was conducted spectrophotometrically following the method described by Velikova et al. (2000). In brief, 5 mL of 0.1% trichloroacetic acid (TCA) was added to 0.5 g of the leaf sample, which was then crushed in a mortar. The resulting homogenate was centrifuged at 10000 *g* for 20 min. To the obtained supernatant, 0.5 mL of 10 mM potassium phosphate buffer (pH 7.0) and 1 mL of 1 M potassium iodide (KI) were added. The supernatants were then measured at 390 nm using a spectrophotometer. The results were compared with a standard graph and reported as μmol g^−1^ FW H_2_O_2_.

For proline analysis, 0.5 g of a fresh leaf sample was digested with 3% sulfosalicylic acid and filtered. From the filtered sample, 2 mL was taken and mixed with 2 mL of acetic acid and 2 mL of ninhydrin reagent (a mixture of ninhydrin, acetic acid, and orthophosphoric acid). The samples were placed in test tubes and incubated in a water bath at 100°C for 1 h. After this incubation, the reaction was halted by placing the samples on ice. Next, 4 mL of toluene was added to the cooled samples, vortexed, and the supernatant part was separated and measured at 520 nm using a spectrophotometer. Calculations were then performed using proline standards processed by the same procedure (Bates et al., 1973).

The determination of malondialdehyde (MDA), a product of lipid peroxidation, was conducted using the method described by Lutts et al. (1996). The preparation of samples for chlorophyll analysis and all procedures performed prior to storage at −80°C were maintained during the MDA analysis. To prepare the samples, 200 mg of the leaf material was taken from those stored in the freezer. Then, 5 mL of 0.1% trichloroacetic acid (TCA) was added to the sample, and the mixture was centrifuged at 10000 *g* for 20 min. After centrifugation, 3 mL of the supernatant was carefully transferred, and an additional 3 mL of 0.1% TCA containing 20% thiobarbituric acid was added. The mixture was then placed in a 95°C hot water bath for 30 min. Finally, the absorbance values were measured using a spectrophotometer at wavelengths of A_532_ and A_600_ nm.

#### 2.2.5 Determination of total phenolics, antioxidants, anthocyanin, carbohydrate and protein

The total phenolic (TPC) content was determined by modifying the method described by Waterhouse (2002). In brief, 1600 mL of pure water and 50 μL of Folin–Ciocalteu reagent were added to 50 μLof the methanolic extract and mixed using a vortex. Next, 300 μL of a 7% sodium carbonate solution were added, and the samples were vortexed again. They were then incubated in the dark for 2 h. After incubation, readings were taken at 760 nm, and the absorbances obtained were converted to real amounts using a standard curve of gallic acid prepared by the same procedure.

To determine the total carbohydrates, a 0.2 mL aliquot of the metabolic extract supernatant was mixed with 0.1 mL of a 5% aqueous phenol solution in a test tube. After, 1 mL of concentrated sulfuric acid was added rapidly to the mixture. The test tubes were allowed to stand for 10 min before being vortexed for 30 s. The samples were then placed in a water bath at room temperature for 20 min to allow for colour development. After this period, the light absorption at 490 nm was measured using a spectrophotometer. Reference solutions were prepared in the same manner, with glucose used as a control that was serially diluted from a 20% concentration (Geater and Fehr, 2000).

The total amount of soluble protein in leaf tissues was determined using the method established by Bradford (1976). Fresh leaf samples were ground in liquid nitrogen and placed in tubes, to which 1.5 mL of KH_2_PO_4_ buffer (pH 7) was added. The samples were then centrifuged at 11000 *g* at +4°C for 20 min. From the resulting supernatant, 20 μL was transferred into a new tube, followed by the addition of 480 μL of distilled water and 5000 μL of Bradford solution. This mixture was vortexed, and the absorbance was measured spectrophotometrically at a wavelength of 595 nm. The total soluble protein content in the leaf tissues was calculated using a standard curve prepared with bovine serum albumin (BSA).

The free radical scavenging activity of 1,1‐diphenyl‐2‐picrylhydrazyl (DPPH) in the samples was assessed using the DPPH method proposed by Güler et al. (2023). To conduct the test, 2.9 mL of a 0.1 mM DPPH solution was added to 0.1 mL of extracts diluted to 1:5 with ethanol. The absorbance of the mixture was measured at 517 nm after 15 min. The DPPH radical scavenging activity of each sample was expressed as a percentage (%) using the formula provided below.

DPPH Inhibition (%) = [(Ac‐As)/Ac × 100].

Ac; absorbance of control, As; absorbance of samples.

To determine the total anthocyanin content, 500 μL of the methanolic extract was mixed with 1500 μL of a methanol/water/HCl (70/30/1 v/v/v) solution. Readings were taken at a wavelength of 540 nm, using the 70/30/1 mixture as a blank. The results were calculated with the following formula and presented as malvidin‐3‐glucoside equivalence.

TA = A_540_ × 16.7 × d.

TA: total anthocyanin, A: absorbance, d: dilution.

#### 2.2.6 Mineral element analysis

The first three samples collected from the tip were dried at 65°C until they reached a constant weight. After drying, the samples were ground using a plant grinding mill. Next, 0.5 g of each sample was burned at 500°C, following the method outlined by Kacar and İnal (2008). To the resulting ash, 4 mL of a 3 N HCl solution was added to obtain extracts. The nutrient element contents (P, K, Ca, Mg, Mn, Fe, Cu, Zn) in the roots and leaves of the prepared extracts were then determined using an ICP‐OES device.

#### 2.2.7 Experimental design and statistical evaluations

The study employed a randomized plot design consisting of three replications with 15 plants in each plot. The data collected was analyzed using a one‐way analysis of variance (ANOVA). Fisher's LSD test was then applied to compare means where the F value indicated statistical significance. Statistical analyses and graphics were generated using the JMP 16 (SAS).

## 3 RESULTS

### 3.1 Growth parameters

Root numbers significantly varied across treatments (*p* ≤ 0.05). In the control group, the average root count was recorded at 7.88. However, with the application of salt, this number decreased to an average of 5.38. In the NaCl + P100 group, the average root count was 4.50, while in the NaCl + P150 group, it increased to an average of 5.00. In contrast, the average root count in the NaCl + P200 group returned to 4.50.

The root length did not possess a significant difference according to the treatments. However, the average root length in the control group was measured at 10.31 cm, whereas this value decreased to an average of 8.78 cm following the salt application. In the NaCl + P100 group, the average root length was found to be 10.13 cm, while in the NaCl + P150 group, it reached an average of 10.63 cm. However, in the NaCl + P200 group, the average root length reverted back to 10.13 cm.

The shoot number was significantly altered by the treatment groups (*p* ≤ 0.05). In the control group, the average leaf count was determined to be 4.88. After the salt application, this number decreased to an average of 4.38. In the NaCl + P100 group, the average leaf count rose to 4.50, while in the NaCl + P150 group, it significantly increased to an average of 5.25. However, in the NaCl + P200 group, the average leaf count dropped to 4.25 (Table 1).

**TABLE 1:** Changes in root number, root length and leaf number in Albion strawberry variety under salt stress as a result of putrescine applications.

Treatments	Root number (pieces)	Root length (cm)	Shoot number (pieces)
Control	7,880,52a	10,31 ± 0,47a	4,88 ± 0,23ab
Salt	5,380,38b	8,78 ± 0,45a	4,38 ± 0,18b
Salt+P100	4,500,38b	10,13 ± 1,03a	4,50 ± 0,33ab
Salt+P150	5,000,46b	10,63 ± 0,63a	5,25 ± 0,31a
Salt+P200	4,500,42b	10,13 ± 0,54a	4,25 ± 0,31b

Different letters in the same column indicate significant differences at *p* ≤ 0.05 according to Fisher's LSD test.

Fresh root weight was significantly affected by the treatments (*p* ≤ 0.05). In the control group, the average fresh root weight was measured at 4.05 g. This value slightly decreased to 4.00 g under salt stress. With the application of putrescine at 100 ppm (NaCl + P100), the average fresh root weight further declined to 3.24 g. At 150 ppm (NaCl + P150), the average fresh root weight decreased to 2.89 g, but a slight recovery was observed at 200 ppm (NaCl + P200), with an average of 3.19 g.

The dry root weight slightly diverged across the treatments, but the differences were not significant. The average dry root weight for the control group was 0.62 g, and this value remained stable under salt stress at 0.59 g. In the NaCl + P100 group, the average dry root weight recorded was 0.52 g. A decrease was also noted in the NaCl + P150 group, where the average was 0.45 g. However, in the NaCl + P200 group, the dry root weight slightly increased to 0.50 g.

The fresh leaf weight varied slightly among the treatments, but these differences were not significant. The control group demonstrated an average fresh leaf weight of 3.66 g, which dropped to 3.35 g under salt stress. After the application of putrescine at 100 ppm (NaCl + P100), the average fresh leaf weight was recorded at 3.30 g. Conversely, the NaCl + P150 group showed a notable increase, reaching an average of 3.85 g, but this value decreased to 2.91 g in the NaCl + P200 group.

The variation among the treatments in dry leaf weights was also insignificant. In the control group, the average dry leaf weight was 0.77 g. Under salt stress, this weight slightly decreased to 0.75 g. The addition of putrescine at 100 ppm (NaCl + P100) maintained the average dry leaf weight at 0.75 g. However, in the NaCl + P150 group, there was an increase to 0.85 g, while the average dry leaf weight in the NaCl + P200 group dropped to 0.65 g (Table 2).

**TABLE 2:** Changes in root, leaf fresh and dry weight (g/plant) of Albion strawberry variety under salt stress with putrescine applications.

Treatments	Fresh root weight (g)	Dry root weight (g)	Fresh leaf weight (g)	Dry leaf weight (g)
Control	4,05 ± 0,39a	0,62 ± 0,06a	3,66 ± 0,29a	0,77 ± 0,05a
NaCl	4,00 ± 0,53a	0,59 ± 0,10a	3,35 ± 0,29a	0,75 ± 0,05a
NaCl+P100	3,24 ± 0,30ab	0,52 ± 0,06a	3,30 ± 0,31a	0,75 ± 0,07a
NaCl +P150	2,89 ± 0,19b	0,45 ± 0,03a	3,85 ± 0,42a	0,85 ± 0,09a
NaCl +P200	3,19 ± 0,38ab	0,50 ± 0,04a	2,91 ± 0,52a	0,65 ± 0,11a

Different letters in the same column indicate significant differences at *p* ≤ 0.05 according to Fisher's LSD test.

### 3.2 Divergence in the Chlorophyll and carotenoid content

**FIGURE 1:**
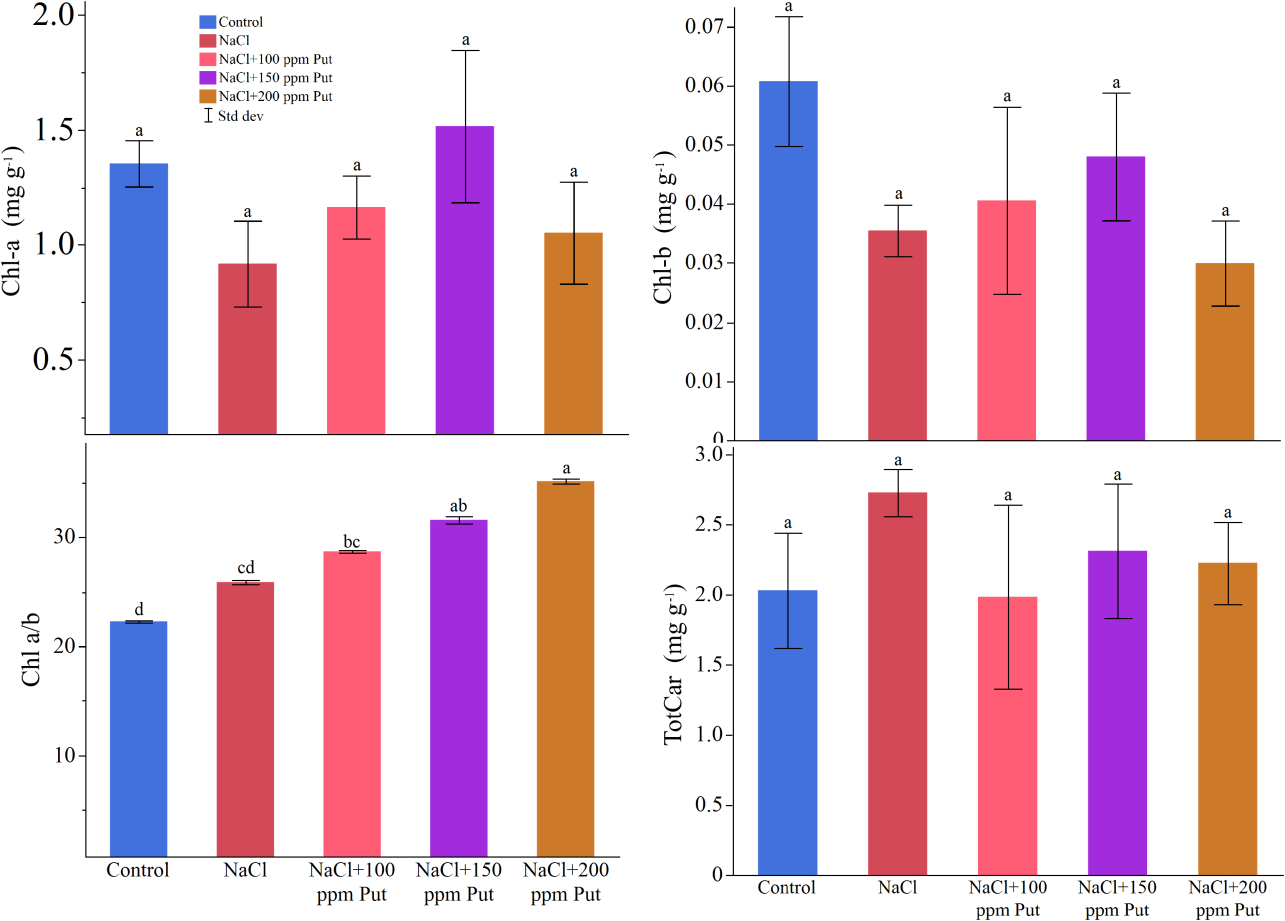
Changes in chlorophyll and carotenoid contents based on the treatments. Different letters on the top of the bars indicate significant differences at *p* ≤ 0.05 according to Fisher's LSD test. Chl: chlorophyll, TotCar: total carotenoids.

The ANOVA results indicated no significant differences among the treatments at *p* ≤ 0.05. The average chlorophyll‐a content in the control group was 1.35 mg g^−1^. The salt treatment averaged 0.92 mg g^−1^, while the NaCl + P100 treatment recorded an average of 1.16 mg g^−1^. The NaCl + P150 treatment showed a higher average of 1.52 mg g^−1^, and the NaCl + P200 treatment averaged 1.05 mg g^−1^ (Figure 1).

The chlorophyll b content did not vary significantly across the treatments. The control group had an average chlorophyll b level of 0.06 mg g^−1^, while the salt treatment averaged 0.04 mg g^−1^. The NaCl + P100 treatment maintained an average of 0.04 ± 0.03 mg g^−1^, the NaCl + P150 treatment had an average of 0.05 mg g^−1^, and the NaCl + P200 treatment averaged 0.03 mg g^−1^ (Figure 1).

Significant differences were noted in the chlorophyll a/b ratio at *p* ≤ 0.05. The control had an average ratio of 22.27, which was significantly higher than the salt treatment's average of 25.87. The NaCl + P100 treatment had an average of 28.67, showing no significant difference from the salt and control groups. In contrast, the NaCl + P150 treatment presented a significantly higher average of 31.56, while the NaCl + P200 treatment had the highest value at 35.10, indicating a significant increase compared to all other treatments (Figure 1).

The carotenoid content was not significantly altered by the treatments (*p* ≥ 0.05). The plants in the control group had an average of 2.03 mg g^−1^, whereas the salt treatment averaged 2.73 mg g^−1^ of carotenoids. The NaCl + P100 treatment decreased carotenoid content to 1.98 mg g^−1^, while the NaCl + P150 treatment resulted in 2.31 mg g^−1^, the highest level observed. The NaCl + P200 treatment possessed a slight decline and averaged 2.23 mg g^−1^ of carotenoids (Figure 1).

### 3.3 Changes in the biochemical constituents

**FIGURE 2:**
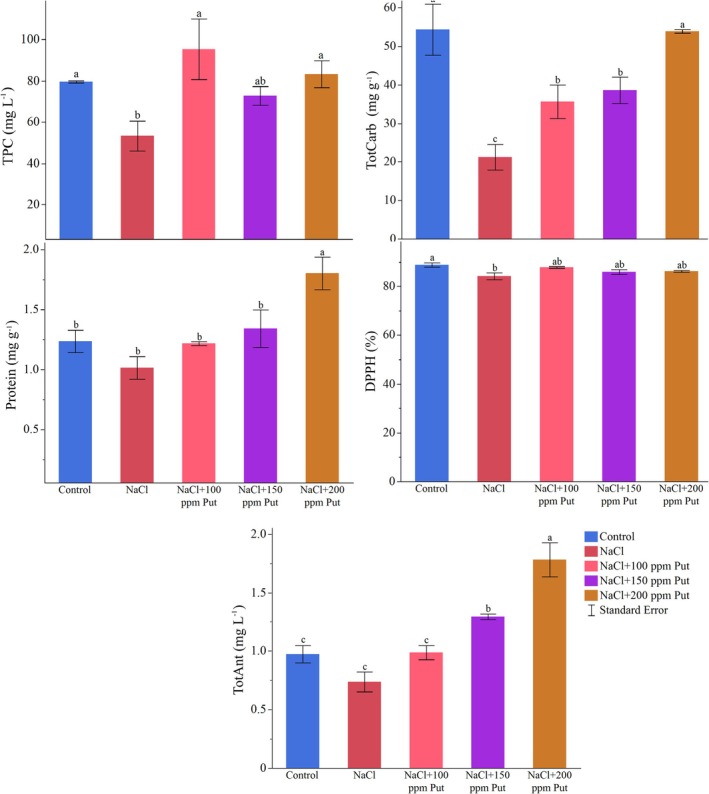
Changes in total phenolic content (TPC), total carbohydrates (TotCarb), protein, DPPH scavenging activity, and total anthocyanin (TotAnt) according to the treatments. Different letters on the top of the bars indicate significant differences at *p* ≤ 0.05 according to Fisher's LSD test.

Measurements of total phenolic content revealed some significant differences at the *p* ≤ 0.05 level. The control group had a total phenolic content of 79.63 mg g^−1^, while the salt group showed a significant decrease to 53.36 mg g^−1^. The NaCl + P100 group recorded an elevated phenolic content of 95.37 mg g^−1^, which is notably high. The NaCl + P150 group had a phenolic content of 72.79 mg g^−1^, while the NaCl + P200 group measured 83.35 mg g^−1^. The phenolic content in the NaCl + P100 group was significantly higher than in the other groups (Figure 2).

Significant differences were noted in total carbohydrate content. The control group had a total carbohydrate level of 54.33 g per 100 g. With salt treatment, this value decreased to 21.22 g per 100 g, demonstrating a significant reduction in carbohydrate content. The carbohydrate content in the NaCl + P100 group was measured at 35.63 g per 100 g, showing a significant decrease compared to the control group but a higher value than the salt group. In the NaCl + P150 group, the total carbohydrate content was 38.59 g per 100 g, which was higher than the salt group but lower than the control group. In the NaCl + P200 group, the total carbohydrate content increased to 53.87 g per 100 g, which is close to the control group's value (Figure 2).

The ANOVA indicated a significant difference between the treatments at the *p* ≤ 0.05 level. In the control group, the total protein content was measured at 1.24 g per 100 g. This value dropped to 1.01 g per 100 g under salt treatment, demonstrating a significant decrease compared to the control group. The total protein content in the NaCl + P100 group was recorded at 1.22 g per 100 g, similar to the control. The protein content was 1.34 g per 100 g in the NaCl + P150. The highest protein content was observed in NaCl + P200, measured at 1.80 g per 100 g, indicating a significant increase compared to the control group (Figure 2).

Salinity and putrescine doses had a significant effect on DPPH scavenging activity (*p* ≤ 0.05). The control group exhibited the highest DPPH scavenging activity at 88.79%, while the lowest was observed with salt application at 84.19%. Notably, a 100 ppm putrescine significantly enhanced DPPH scavenging activity under saline conditions to 87.80% (Figure 2).

Significant differences were observed in total anthocyanin content at *p* ≤ 0.05. The control group had a total anthocyanin level of 0.97 mg g^−1^, while the content decreased to 0.74 mg g^−1^ under saline conditions, indicating a diminish in anthocyanin by salinity stress. The NaCl + P100 group exhibited a total anthocyanin content of 0.99 mg g^−1^, which is significantly higher than that of sole stress conditions. In the NaCl + P150 group, the value was measured at 1.29 mg g^−1^, and the highest value of 1.78 mg g^−1^ was found in the NaCl + P200 group. This indicates that the NaCl + P200 group had a significant increase in anthocyanin content compared to the other groups (Figure 2).

### 3.4 Changes in enzyme metabolism

**FIGURE 3:**
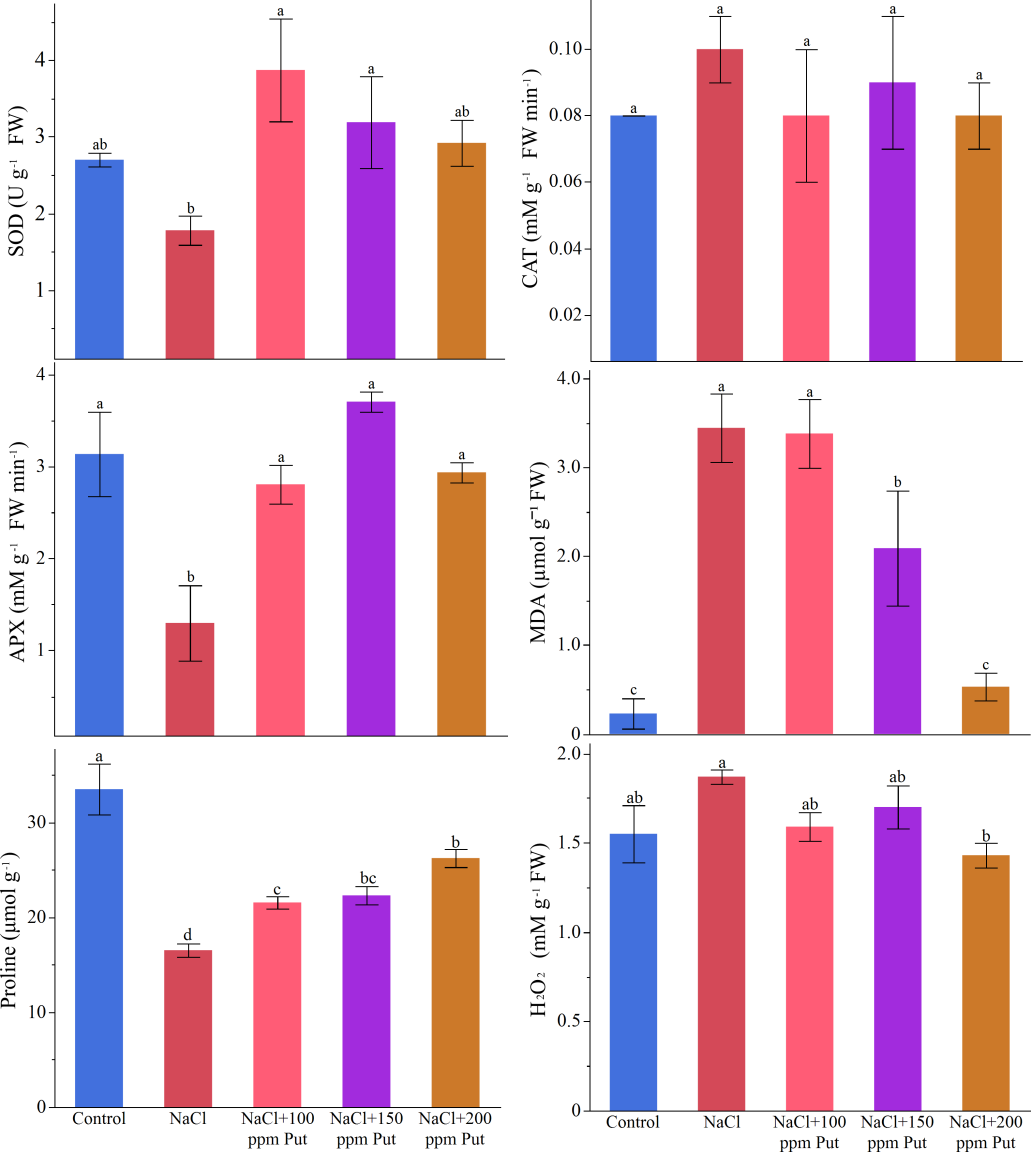
Fluctuations in oxidative and antioxidant enzyme metabolism induced by salinity and putrescine.

SOD activity was significantly influenced by the salinity and putrescine doses (*p* ≤ 0.05). The highest SOD activity was observed in 100 ppm putrescine‐applied strawberry plants under salinity stress (3.87 ± 0.67 U), followed by 150 ppm putrescine (3.19 ± 0.60 U). Notably, SOD activity slightly declined with the putrescine dose increment, but it was still higher than in saline and non‐stress conditions. The lowest activity was measured under saline stress with an average of 1.78 U. CAT activity fluctuated slightly across treatments, but the difference was insignificant (*p* > 0.05). CAT activities varied between 0.08 mM g FW/min (Control, P100, and P200) and 0.10 mM g^−1^ FW min^−1^ (1 g L^−1^ NaCl). APX activity significantly fluctuated among treatments (*p* ≤ 0.05). The salt‐stressed strawberry plants possessed the lowest APX activity with a mean of 1.30 mM g^−1^ FW min^−1^ value, while P150 exhibited the highest activity at 3.71 mM g^−1^FW min^−1^. Salt‐stressed plants sprayed with 100 and 200 ppm putrescine exhibited similar APX activities, measuring 2.81 and 2.94 mM g^−1^ FW min^−1^ activities, respectively. MDA activity was strikingly induced by salt stress, with a 0.23 μM g^−1^FW activity in control and a 3.44 μM g^−1^FW in the saline condition. The rising putrescine doses progressively suppressed MDA activity, which was 3.38, 2.09, and 0.53 μM g^−1^ FW in 100, 150 and 200 ppm, respectively. Proline content significantly varied across strawberry plants treated with saline and putrescine doses (*p* ≤ 0.05). Proline was progressively improved by increasing putrescine doses and reached from 16.53 μM g^−1^ FW‐(saline stress) to 21.56 μM g^−1^ FW in 100 ppm, to 22.30 μM g^−1^ FW in 150 ppm, and to 26.23 μM g^−1^ FW in 200 ppm putrescine treatments. The H_2_O_2_ activity was significantly affected by the treatments (*p* ≤ 0.05). The plants grown under saline conditions possessed the highest H_2_O_2_ activity (1.87 mM g^−1^ FW), while 200 ppm putrescine treatment lowered it to a mean of 1.43 mM g^−1^ FW. Although the other groups were not significantly different, nonstressed plants exhibited a lower activity of H_2_O_2_ (1.55 mM g^−1^ FW) (Figure 3).

### 3.5 Plant nutrient contents

**FIGURE 4:**
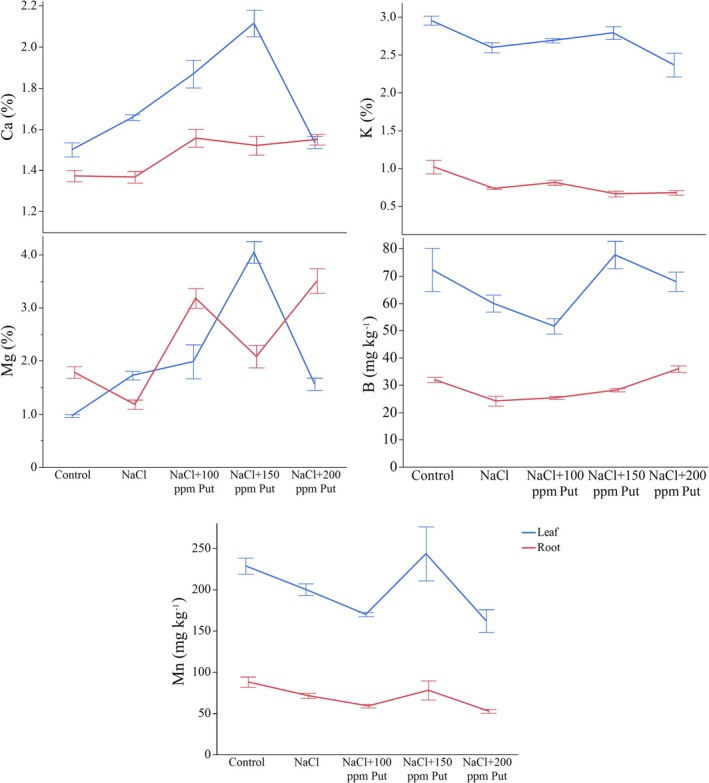
Alterations of Ca, K, Mg, Mn, and B according to salt and putrescine treatments in hydroponic Albion strawberries.

The ANOVA results indicate that the effects of tissue type (leaf and root), treatments as well as their interaction on calcium content were significant (*p* ≤ 0.001). In the leaves, the calcium content increased from 1.50% in the control group to 1.66% under salt stress. The content rose to 1.87% with the 100 ppm putrescine treatment under NaCl stress. The highest calcium content observed was 2.12% in the 150 ppm putrescine treatment. However, when 200 ppm putrescine was applied, the calcium content decreased to 1.54%, which is close to the control level. In the roots, the calcium content remained constant at 1.37% for both the control and NaCl treatments. However, the application of putrescine significantly increased root calcium content, and Ca content was measured as 1.56% in NaCl + P100, 1.52% in NaCl + P150, and 1.55% in NaCl + P200 (Table S1, Figure 4).

The effects of tissue type (*p* ≤ 0.001) and treatment (*p* ≤ 0.001) on potassium content were significant, whereas the interaction between tissue type and treatment was not significant (*p* > 0.05). In leaves, the highest potassium content was measured in the control group at 2.96%. Treatment with NaCl reduced the potassium content to 2.60%. Additionally, the application of 100 ppm and 150 ppm putrescine slightly increased the potassium content to 2.69 and 2.79%, respectively. However, the application of 200 ppm putrescine decreased potassium content to 2.37%. In roots, the highest potassium content was also observed in the control group at 1.02%, which significantly declined to 0.73% under the NaCl treatment. The putrescine slightly mitigated this reduction, with 100 ppm resulting in a potassium content of 0.81%, 150 ppm at 0.66%, and 200 ppm at 0.68%. Nevertheless, potassium content in the roots did not return to control levels under any treatment (Table S1, Figure 4).

The effects of tissues (*p* ≤ 0.05) and treatments (*p* ≤ 0.001) on magnesium content were significant. In leaves, magnesium content was lowest in the control group (0.97%). Salt stress increased the magnesium content to 1.73%. The application of 100 ppm putrescine further increased magnesium content to 1.99%, and 150 ppm putrescine resulted in the highest observed value for leaves (4.05%). However, magnesium content decreased to 1.56% with 200 ppm putrescine. In roots, magnesium content in the control group was 1.79% but decreased significantly to 1.18% under NaCl treatment. The application of 100 ppm putrescine increased root magnesium content to 3.18%, while 200 ppm putrescine resulted in the highest root magnesium content (3.51%). The 150 ppm putrescine resulted in 2.08% in root magnesium content (Table S1, Figure 4).

According to the ANOVA results, the effects of tissues and treatments on manganese (Mn) content were significant (*p* ≤ 0.001), while the interaction between tissues and treatments was not significant (*p* > 0.05). In leaves, the Mn content in the control group was 228.74 mg kg^−1^ and decreased to 200.17 mg kg^−1^ under NaCl. The application of 100 ppm putrescine further reduced Mn content to 169.95 mg kg^−1^, which was the lowest Mn level observed in leaves. However, Mn content increased with 150 ppm putrescine to 243.47 mg/kg, which was even higher than the control group. A slight reduction in Mn content was observed with 200 ppm putrescine at 162.06 mg kg^−1^. In roots, the Mn content was highest in the control group (88.09 mg kg^−1^) but decreased to 71.44 mg/kg^−1^ under NaCl alone. Mn content was further reduced with 100 ppm (58.95 mg kg^−1^) and 200 ppm putrescine (52.70 mg kg^−1^), while 150 ppm putrescine resulted in a slightly higher content of 77.93 mg kg^−1^ (Table S2, Figure 4).

In hydroponically grown Albion, the effects of tissues (*p* ≤ 0.001), treatments (*p* ≤ 0.001), and their interaction (*p* ≤ 0.05) on boron (B) content were significant. The B content in the control group was measured as 72.23 mg kg^−1^ but decreased significantly to 59.93 mg kg^−1^ under NaCl treatment in the leaves. The application of 100 ppm putrescine further reduced boron to 51.58 mg kg^−1^, the lowest value observed in leaves. B content recovered significantly to 77.66 mg kg^−1^ with 150 ppm putrescine application, the highest value recorded among all treatments in leaves. At 200 ppm putrescine, B content decreased to 67.89 mg kg^−1^. In roots, B content was recorded as 31.92 mg kg^−1^ in the control group and declined to 24.14 mg kg^−1^ under NaCl stress. A minimal increase was observed at 100 ppm putrescine (25.32 mg kg^−1^), while 150 ppm putrescine increased B content to 28.18 mg kg^−1^. Remarkably, the highest boron content in roots was observed with 200 ppm putrescine treatment at 35.88 mg kg^−1^ (Table S2, Figure 4).

**FIGURE 5:**
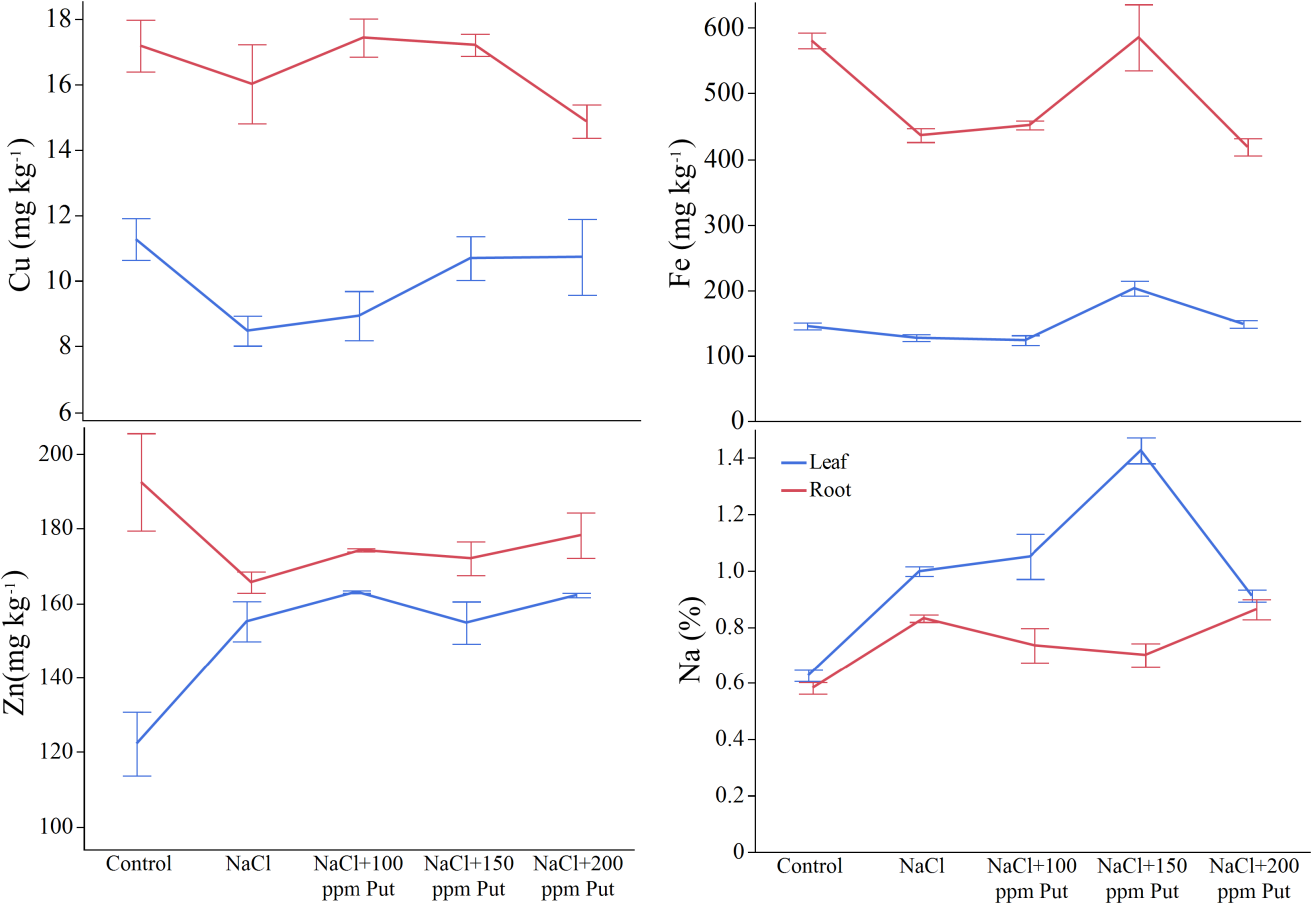
Fluctuations in nutrients; Cu, Fe, Zn, and Na influenced by salt and putrescine treatments in hydroponic Albion strawberries.

The effects of tissues (*p* ≤ 0.001) on copper (Cu) content were significant, but treatment effects and the interaction of tissues and treatments were not significant (*p* > 0.05). The Cu content was highest in the control group at 11.28 mg kg^−1^ but decreased to 8.49 mg kg^−1^ under NaCl treatment in the leaves. With NaCl + P100, Cu content slightly increased to 8.94 mg kg^−1^, and with NaCl + P150 and NaCl + P200, Cu content increased further to 10.70 and 10.74 mg kg^−1^, respectively, closing the control value. In roots, Cu content was similar across treatments, with the highest value recorded as 17.44 mg kg^−1^ under NaCl + P100. In contrast, NaCl + P200 exhibited the lowest Cu content in roots (14.88 mg kg^−1^). Cu was otherwise stable across other treatments, ranging from 16.03 to 17.22 mg kg^−1^ (Table S2, Figure 5).

The ANOVA results indicated that tissue type (*p* ≤ 0.001), treatment (*p* ≤ 0.001), and their interaction (*p* ≤ 0.01) had significant effects on iron (Fe) content. In the leaves, the control group exhibited a Fe content of 145.02 mg kg^−1^, which decreased to 127.20 mg kg^−1^ under NaCl stress. The lowest Fe content in the leaves was recorded with NaCl + P100 at 123.57 mg kg^−1^. Conversely, the application of 150 ppm putrescine significantly increased Fe content to 202.87 mg/kg^−1^, the highest level observed in the leaves. However, the Fe content slightly declined to 148.31 mg kg^−1^ with 200 ppm putrescine treatment. The highest Fe content was in the control group at 581.12 mg kg^−1^, but NaCl stress significantly reduced this amount to 436.51 mg kg^−1^ in the roots of hydroponic Albion strawberry. The application of putrescine under NaCl conditions generally helped maintain Fe content closer to control levels. Notably, 150 ppm putrescine delivered a recovery to 585.69 mg kg^−1^—the highest value among all treatments. Meanwhile, 100 and 200 ppm putrescine treatments resulted in intermediate Fe contents of 451.92 and 418.45 mg kg^−1^, respectively (Table S3, Figure 5).

The ANOVA results showed significant effects of tissues (*p* ≤ 0.001), treatments (*p* ≤ 0.001), and their interaction (*p* ≤ 0.001) on sodium (Na) content. In leaves, Na content in the control group was 0.63% and significantly increased to 1.00% under NaCl stress. The 100 ppm putrescine treatment further increased Na content to 1.05%, while the highest Na content in leaves was recorded with 150 ppm putrescine treatment (1.43%). When 200 ppm putrescine was applied, the Na content decreased slightly to 0.91%. In roots, the Na content in the control group was 0.58%, which increased to 0.83% under NaCl stress. NaCl + P100 slightly reduced the Na content to 0.73%, while NaCl + P150 resulted in a further decrease to 0.70%. However, 200 ppm putrescine (NaCl + P200) led to the highest Na content in roots (0.86%), similar to the level observed under NaCl alone (Table S3, Figure 5).

Zinc (Zn) content was significantly affected by tissues (*p* ≤ 0.001). However, treatment and the interaction of tissues and treatments were not significant (*p* > 0.05). Zn content in the control group was 122.21 mg kg^−1^ in the leaves. Under NaCl stress, Zn content increased significantly to 155.13 mg kg^−1^, and with NaCl + P100 and NaCl + P150, Zn content increased further to 163.10 and 154.80 mg kg^−1^, respectively. NaCl + P200 resulted in a Zn content of 162.18 mg kg^−1^, slightly lower than NaCl + P100. In roots, the control group had the highest Zn content at 192.53 mg kg^−1^ and significantly declined to 165.66 mg kg^−1^ under NaCl stress. Putrescine applications resulted in slight recoveries, with NaCl + P100 (174.31 mg kg^−1^), NaCl + P150 (172.07 mg kg^−1^), and NaCl + P200 (178.27 mg kg^−1^ all increasing Zn content compared to NaCl (Table S3, Figure 5).

## 4 DISCUSSION

Salinity stress primarily impacts the physiological processes of plants, particularly photosynthesis and nutrient uptake. Under saline conditions, the osmotic potential of the soil solution decreases, making it more difficult for plants to absorb water. This results in physiological drought, inhibiting photosynthesis due to reduced leaf turgor and stomatal closure. Consequently, lower carbon dioxide assimilation rates lead to reduced growth (EL‐Metwally, 2023; Kim et al., 2016). The chlorophyll content, crucial for photosynthesis, is significantly affected by salinity. Studies have shown that salinity stress leads to a decrease in chlorophyll a, chlorophyll b, and carotenoid levels in strawberries, directly correlating with reduced photosynthetic efficiency and overall plant vigour (Mozafari et al., 2018; Karakaş et al., 2021). In this study, a notable decrease in chlorophyll content was also observed under saline stress, supporting previous research. However, the decline in plant growth was relatively limited compared to changes in chlorophyll content, most likely due to the timing of sampling. Metabolic effects on morphometric characteristics tend to become more pronounced during extended growth phases, as suggested by Guo et al. (2016) in studies on *Lycium ruthenicum* Murr. plants under drought stress.

Putrescine can stabilize thylakoid membranes and protect chlorophyll content, which is vital for photosynthesis. Islam et al. (2021) reported that exogenous putrescine application significantly increased chlorophyll levels in Korean ginseng sprouts under saline conditions, thereby enhancing photosynthetic performance. This effect is corroborated by findings from Kou et al. (2018), who noted that putrescine helps maintain the integrity of the photosynthetic apparatus by regulating unsaturated fatty acid content and ROS homeostasis. The positive impact of putrescine on chlorophyll metabolism has also been observed in cucumber seedlings, where it promoted the conversion of uroporphyrinogen III to protoporphyrin IX, thereby alleviating decreases in chlorophyll content under salt stress (Yuan et al., 2018). Here, we also report a notable improvement of chlorophyll by foliar putrescine spray in strawberry plants subjected to saline stress. Noteworthily, the enhancement was somewhat linear until 150 ppm declined in 200 ppm treatment, indicating that oversupplied putrescine could harm the chlorophyll metabolism in strawberry plants. The 150 ppm putrescine treatment particularly enhanced chlorophyll‐a content, while the chlorophyll a/b ratio was significantly boasted by increasing putrescine doses. Recent studies emphasize that the chlorophyll a/b ratio is correlated with the structural and functional adaptations of the photosynthetic machinery within plants. Under stress conditions such as salinity or nutrient limitation, chlorophyll b levels often decline more rapidly than chlorophyll‐a, resulting in a higher a/b ratio (Soares et al., 2023; Alharbi et al., 2022). This shift suggests an adaptive response wherein plants alter their pigment composition to optimize photosynthesis under adverse conditions. Such plasticity in chlorophyll ratios can significantly impact plant health and productivity (Osu et al., 2020; Ali et al., 2022). The improved chlorophyll a/b ratio from putrescine in this study indicates that applying putrescine to the leaves could enhance photosynthesis efficiency and, consequently, promote plant growth under salt stress. The enhancement in the chlorophyll composition by putrescine was also supported by the improved growth, particularly in the 150 ppm treatment, which yielded higher leaf growth.

Under salinity stress, the normal metabolism of plant cells becomes disrupted, leading to the excessive production of reactive oxygen species (ROS) and resulting in oxidative stress. Under these conditions, ROS can damage chlorophyll, proteins, and cell membranes (Ghanbari et al., 2023). To combat oxidative stress, plants increase their levels of antioxidants. Phenolic compounds are recognized for their antioxidant properties, and an enhanced synthesis of these compounds leads to improved antioxidant activity (Valifard et al., 2017). One of the main mechanisms by which putrescine alleviates salinity stress is through its impact on antioxidant and phenolic metabolism. Ghosh et al. (2012) demonstrated that treating rice with putrescine resulted in increased levels of non‐enzymatic antioxidants such as flavonoids and anthocyanins under salt stress, which helped reduce oxidative damage and improve plant health. In this study, the contents of phenolics, anthocyanins, proline, and antioxidants were significantly enhanced by putrescine treatments in the Albion cultivar under saline conditions. This suggests that putrescine could serve as a universal stress‐relief agent across the plant kingdom by promoting the metabolism of phenolics and antioxidants.

The physiological responses to salinity stress also involve the production of osmoprotectants, such as proline and glycine betaine, which help stabilize cellular structures and protect against oxidative damage (Rodrigues Neto et al., 2023). These compounds play a crucial role in osmotic adjustment, allowing plants to maintain turgor pressure and cellular integrity in the face of high salinity (Rodrigues Neto et al., 2023; Heidari, 2011). The accumulation of these osmolytes is often correlated with enhanced stress tolerance, as they mitigate the adverse effects of ionic stress and protect cellular functions (Heidari, 2011). In addition to osmoprotectants, the production of ROS is a common response to salinity stress. While excessive ROS can lead to oxidative damage, plants have evolved antioxidant defence mechanisms to counteract this effect (Kim et al., 2022). The upregulation of antioxidant enzymes, such as SOD and CAT, is often observed in plants subjected to salinity stress, providing a protective mechanism against oxidative damage (Riyazuddin et al., 2020). This balance between ROS production and antioxidant activity is crucial for maintaining cellular homeostasis and promoting stress tolerance. Numerous studies indicate that exogenous application of putrescine enhances antioxidant enzyme activities in plants subjected to salinity. Research demonstrates that putrescine increases the activity of SOD, APX, and glutathione peroxidase (GPX), which are crucial in detoxifying ROS (Xiong et al., 2018; Gohari et al., 2021; Ghosh et al., 2012). Additionally, putrescine treatment has been associated with elevated levels of non‐enzymatic antioxidants such as proline and phenolic compounds, which act synergistically to mitigate oxidative damage and enhance plant resilience to salinity (Jalili et al., 2023; El‐Badri et al., 2021). In this study, the increased enzymatic activity of SOD and CAT, along with osmolytes such as proline induced by putrescine treatments, indicates a beneficial effect on ROS scavenging in strawberries under saline conditions.

High salinity levels lead to osmotic stress, which restricts water absorption and creates a physiological drought condition, ultimately impairing the plant's ability to absorb essential nutrients such as K, Ca, and Mg (Khan, 2024; Pan et al., 2022; Lü et al., 2021). The disruption of nutrient uptake mechanisms is compounded by the toxic effects of Na and Cl ions, which can accumulate to harmful levels within plant tissues, further exacerbating nutrient imbalances (Balliu et al., 2015; Idder et al., 2019; Niu et al., 2012). The impact of putrescine and salt stress on mineral uptake and plant composition is gaining more attention in plant physiology, particularly due to the role of putrescine as a natural polyamine that can modulate plant responses to abiotic stress. Exogenous application of putrescine under salt stress conditions has been shown to improve growth parameters in several plant species, likely due to its effect on mineral nutrient accumulation and antioxidant activity. Putrescine treatments have been shown to enhance the uptake of mineral nutrients in Brassica juncea under saline conditions (Lakra et al., 2006). Garg and Sharma (2019) reported that the application of 1 mM putrescine to pigeonpea (*Cajanus cajan* L.) under saline conditions resulted in increased levels of phosphorus, Ca^+^/Na^+^, and K^+^/Na^+^. Additionally, Mansour et al. (2024) observed significant improvements in N, P, K, and Mg levels in Jatropha plants subjected to 5000 ppm saline conditions. In strawberries, Keutgen et al. (2008) reported slight increases in mineral levels such as K^+^, N, P, and Zn^2+^, along with substantial increases in Na^+^ and Cl^−^ levels due to salinity. Conversely, Yildirim et al. (2009) found that 40 mM NaCl led to significant reductions in the levels of macronutrients in strawberries. We observed decreased levels of Zn^2+^, Fe, Cu, B, Mn, and K in Albion strawberries under 1000 ppm salt stress. The changes in mineral content were tissue‐specific, with notably higher concentrations of heavy metals found in the roots. Treatment with putrescine generally improved the nutrient status in both the roots and leaves, with the exception of Mn. The similarities and differences observed in previous research may be attributed to the applied doses and genetic variations among the plants. Ferreira et al. (2019) found non‐significant variations in the mineral nutrient contents of the leaves, roots, and petioles of the Albion and Benicia cultivars. In contrast, the Monterey, San Andreas, and Ventana cultivars exhibited significant fluctuations in nutrient levels, which were influenced by the varying doses across different tissues. Considering this and previous research, strawberries respond to salt stress in a dose‐ and genotype‐dependent manner; however, the beneficial effects of putrescine appear to be consistent across various doses and cultivars.

## 5 CONCLUSION

**FIGURE 6:**
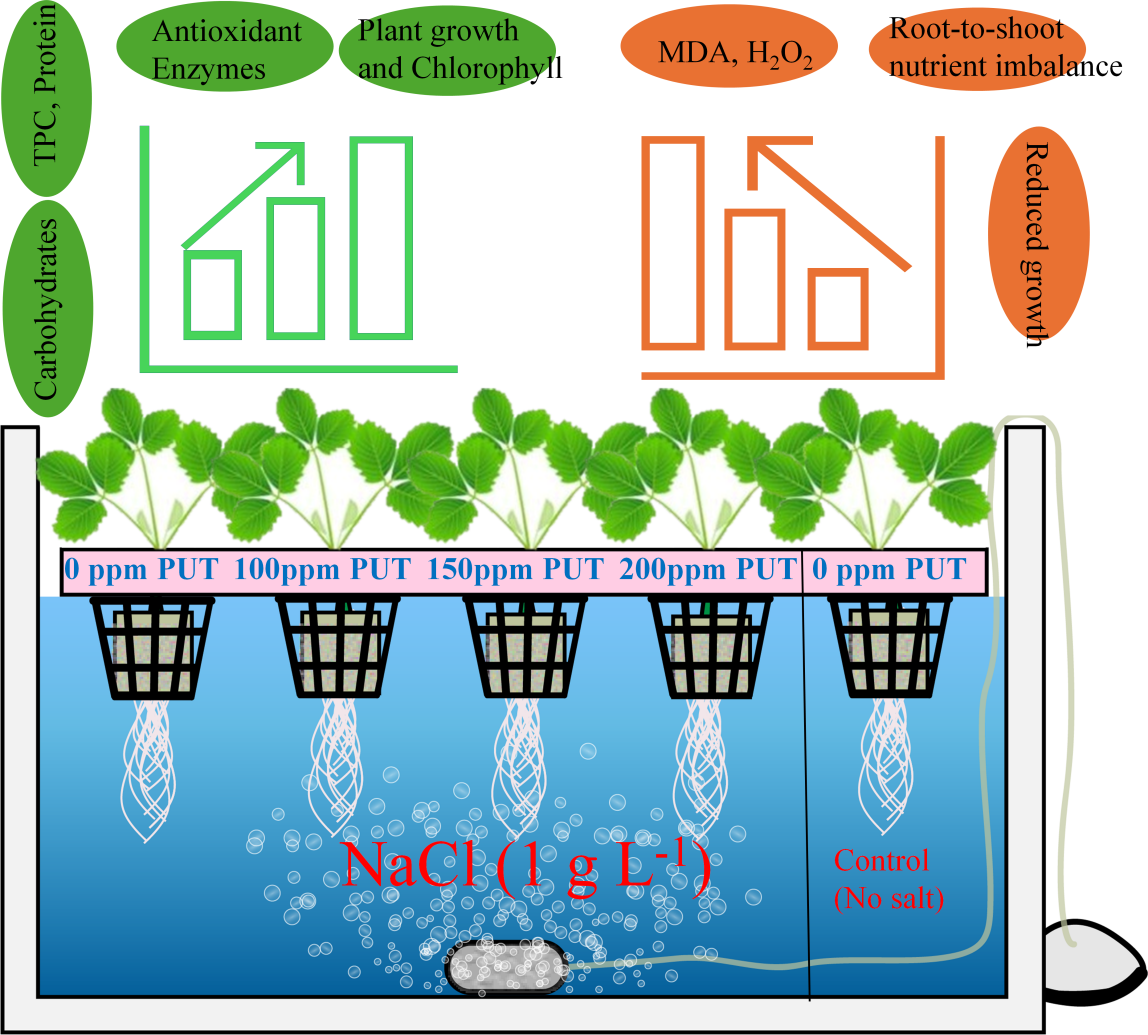
The schematized effect mechanism of salt‐induced stress and the ameliorative effect of putrescine in strawberry (cv. Albion) in hydroponic culture.

This study demonstrates that exogenous application of putrescine can effectively alleviate the adverse effects of salinity stress. Putrescine treatments contribute to improved plant resilience in strawberry (cv. Albion) by stabilizing chlorophyll content, enhancing antioxidant enzyme activities, and regulating nutrient balance. However, our findings underscore the importance of optimizing putrescine concentration, as excessive application may negatively impact chlorophyll metabolism. A schematized mechanism was provided in Figure 6. Overall, the insights from this study provide a comprehensive understanding of the physiological and biochemical responses of strawberries to salinity stress, offering practical applications for improving crop resilience. Further research should focus on fine‐tuning these interventions across diverse environmental conditions and integrating them into holistic management strategies to address the challenges of salinity in agriculture.

## AUTHOR CONTRIBUTIONS

Ferhad Muradoğlu: Writing‐original draft, Formal analysis, Data curation, Methodology, Project administration, Supervision. Şeyma Batur: Writing‐original draft, Formal analysis, Data curation, Investigation. Mirmahmud Hasanov: Data curation, Visualization, Investigation. Emrah Güler: Writing‐review & editing, Investigation, Visualization, Writing original draft.

## CONFLICT OF INTEREST STATEMENT

The authors declare that they have no known competing financial interests or personal relationships that could have appeared to influence the work reported in this work.

## FUNDING & ACKNOWLEDGEMENTS

This manuscript includes parts of the MSc thesis of Mirmahmud Hasanov carried out under the supervision of Ferhad Muradoğlu. The study was financed by the Scientific Research Projects unit of Bolu Abant Izzet Baysal University with the grant number of 2022.10.05.1573. The authors express special thanks to ÇİLTAR® and its manager, Mr. Ali Türemiş, for providing the strawberry plant materials used in this study.

## Supporting information


**Data S1:** Supporting Information

## Data Availability

The data used for this research is available from the corresponding author upon request.
