# Supplementary material for: Putrescine eases saline stress by regulating biochemicals, antioxidative enzymes, and osmolyte balance in hydroponic strawberries (cv. Albion)
Source: Physiol Plant. 2025 May 9;177(3):e70259. doi: 10.1111/ppl.70259 (PMC12062852; doi:10.1111/ppl.70259)
Supplement: Supplementary file 1 — Data S1: Supporting Information [file PPL-177-e70259-s001.docx]

**Putrescine eases saline stress by regulating biochemicals, antioxidative enzymes, and osmolyte balance in hydroponic strawberries (cv. Albion)**

Ferhad Muradoğlu^1*^, Şeyma Batur^1^, Mirmahmud Hasanov^2^, Emrah Güler^1^

^1^ Department of Horticulture, Faculty of Agriculture, Bolu Abant Izzet Baysal University, Bolu 14030, Türkiye

^2^ Institute of Graduate Education, Faculty of Agriculture, Bolu Abant Izzet Baysal University, Bolu 14030, Türkiye

**Supplemantary Tables**

**Supp. Table 1.** Effects of exogenous putrescine applications on Ca, K and Mg nutrient element changes in root and leaf of Albion strawberry variety under salt stress.

| **Tissue** | **Treatment** | **Ca (%)** | **K (%)** | **Mg (%)** |
| --- | --- | --- | --- | --- |
| Leaf | Control | 1.50 ± 0.03 d | 2.96 ± 0.06 a | 0.97 ± 0.03 c |
|  | NaCl | 1.66 ± 0.01 c | 2.60 ± 0.07 bc | 1.73 ± 0.08 b |
|  | NaCl-Put100 | 1.87 ± 0.07 b | 2.69 ± 0.03 ab | 1.99 ± 0.32 b |
|  | NaCl-Put150 | 2.12 ± 0.06 a | 2.79 ± 0.08 ab | 4.05 ± 0.20 a |
|  | NaCl-Put200 | 1.54 ± 0.03 cd | 2.37 ± 0.16 c | 1.56 ± 0.12 b |
|  |  |  |  |  |
| Root | Control | 1.37 ± 0.03 b | 1.02 ± 0.09 a | 1.79 ± 0.11 b |
|  | NaCl | 1.37 ± 0.03 b | 0.73 ± 0.01 b | 1.18 ± 0.09 c |
|  | NaCl-Put100 | 1.56 ± 0.04 a | 0.81 ± 0.03 b | 3.18 ± 0.19 a |
|  | NaCl-Put150 | 1.52 ± 0.05 a | 0.66 ± 0.04 b | 2.08 ± 0.21 b |
|  | NaCl-Put200 | 1.55 ± 0.03 a | 0.68 ± 0.03 b | 3.51 ± 0.23 a |
| **ANOVA** | | |  |  |
| *F*_Tissue_ | | 100.01*** | 1741.60*** | 6.60* |
| *F*_Treatment_ | | 28.43*** | 10.98*** | 35.46*** |
| *F*_Tissue x Treatment_ | | 15.11*** | 2.41ns | 37.96*** |

Data were represented as Mean ± standard error. Different letters in the same column for each tissue indicate significant differences at p≤0.05 according to Fisher’s LSD test. * and *** indicate significance at p≤0.05 and p≤0.001, respectively. ns: non-significant.

**Supp. Table 2.** Effects of exogenous putrescine application on the changes in Mn, B, and Cu nutrient elements in the roots and leaves of the Albion strawberry variety under salt

| **Tissue** | **Treatment** | **Mn (mg/kg)** | **B (mg/kg)** | **Cu (mg/kg)** |
| --- | --- | --- | --- | --- |
| Leaf | Control | 228.74 ± 9.77 a | 72.23 ± 7.90 ab | 11.28 ± 0.64 a |
|  | NaCl | 200.17 ± 7.07 ab | 59.93 ± 3.12 bc | 8.49 ± 0.46 b |
|  | NaCl-Put100 | 169.95 ± 2.70 b | 51.58 ± 2.82 c | 8.94 ± 0.75 ab |
|  | NaCl-Put150 | 243.47 ± 32.82 a | 77.66 ± 5.01 a | 10.70 ± 0.67 ab |
|  | NaCl-Put200 | 162.06 ± 13.94 b | 67.89 ± 3.58 ab | 10.74 ± 1.16 ab |
|  |  |  |  |  |
| Root | Control | 88.09 ± 6.13 a | 31.92 ± 0.95 b | 17.19 ± 0.79 ab |
|  | NaCl | 71.44 ± 3.16 abc | 24.14 ± 1.79 d | 16.03 ± 1.21 ab |
|  | NaCl-Put100 | 58.95 ± 2.13 bc | 25.32 ± 0.56 cd | 17.44 ± 0.58 a |
|  | NaCl-Put150 | 77.93 ± 11.53 ab | 28.18 ± 0.60 c | 17.22 ± 0.34 ab |
|  | NaCl-Put200 | 52.70 ± 2.30 c | 35.88 ± 1.23 a | 14.88 ± 0.51 b |
| **ANOVA** | | |  |  |
| *F*_Tissue_ | | 265.92*** | 272.09*** | 184.43*** |
| *F*_Treatment_ | | 7.41*** | 7.30*** | 2.30ns |
| *F*_Tissue x Treatment_ | | 1.67ns | 3.10* | 2.38ns |

Data were represented as Mean ± standard error. Different letters in the same column for each tissue indicate significant differences at p≤0.05 according to Fisher’s LSD test. *** indicate significance at p≤0.001. ns: non-significant.

**Supp Table 3.** Effects of exogenous putrescine applications on Fe, Na and Zn nutrient element changes in root and leaf of Albion strawberry variety under salt stress.

| **Tissue** | **Treatment** | **Fe (mg/kg)** | **Na (%)** | **Zn (mg/kg)** |
| --- | --- | --- | --- | --- |
| Leaf | Control | 145.02 ± 5.26 bc | 0.63 ± 0.02 d | 122.21 ± 8.58 b |
|  | NaCl | 127.20 ± 5.27 bc | 1.00 ± 0.02 bc | 155.13 ± 5.42 a |
|  | NaCl-Put100 | 123.57 ± 7.52 c | 1.05 ± 0.08 b | 163.10 ± 0.37 a |
|  | NaCl-Put150 | 202.87 ± 11.18 a | 1.43 ± 0.05 a | 154.80 ± 5.67 a |
|  | NaCl-Put200 | 148.31 ± 5.83 b | 0.91 ± 0.02 c | 162.18 ± 0.63 a |
|  |  |  |  |  |
| Root | Control | 581.12 ± 11.91 a | 0.58 ± 0.02 d | 192.53 ± 13.09 a |
|  | NaCl | 436.51 ± 10.60 b | 0.83 ± 0.01 ab | 165.66 ± 2.87 b |
|  | NaCl-Put100 | 451.92 ± 6.73 b | 0.73 ± 0.06 bc | 174.31 ± 0.42 ab |
|  | NaCl-Put150 | 585.69 ± 50.51 a | 0.70 ± 0.04 cd | 172.07 ± 4.51 ab |
|  | NaCl-Put200 | 418.45 ± 13.08 b | 0.86 ± 0.04 a | 178.27 ± 6.09 ab |
| **ANOVA** | | |  |  |
| *F*_Tissue_ | | 905,63*** | 99,54*** | 42,20*** |
| *F*_Treatment_ | | 16,99*** | 32,49*** | 1,58ns |
| *F*_Tissue x Treatment_ | | 6,41** | 23,60*** | 8,69*** |

Data were represented as Mean ± standard error. Different letters in the same column for each tissue indicate significant differences at p≤0.05 according to Fisher’s LSD test. ** and *** indicate significance at p≤0.01 and p≤0.001, respectively. ns: non-significant.
